# Cohort protocol paper: The Pain and Opioids In Treatment (POINT) study

**DOI:** 10.1186/2050-6511-15-17

**Published:** 2014-03-20

**Authors:** Gabrielle Campbell, Richard Mattick, Raimondo Bruno, Briony Larance, Suzanne Nielsen, Milton Cohen, Nicholas Lintzeris, Fiona Shand, Wayne D Hall, Bianca Hoban, Chyanne Kehler, Michael Farrell, Louisa Degenhardt

**Affiliations:** 1 National Drug and Alcohol Research Centre, UNSW, Sydney, NSW 2052, Australia; 2 School of Medicine, University of Tasmania, Hobart, Tasmania, Australia; 3 Discipline of Addiction Medicine, The University of Sydney, Sydney, Australia; 4 The Langton Centre, South East Sydney Local Health District (SESLHD) Drug and Alcohol Services, Surry Hills, Australia; 5 St Vincent’s Clinical School, UNSW Medicine, UNSW, Darlinghurst, Australia; 6 Black Dog Institute, UNSW, Randwick, Australia; 7 Centre for Youth Substance Abuse Research, University of Queensland, Queensland, Australia; 8 National Addiction Centre, Kings College, London, England; 9 School of Population and Global Health, University of Melbourne, Melbourne, Australia; 10 Centre for Adolescent Health, Murdoch Children’s Research Institute, Melbourne, Australia; 11 Department of Global Health, School of Public Health, University of Washington, Seattle, USA

**Keywords:** Opioids, Chronic non-cancer pain, Pain, Quality of life, Dependence, Non-adherence

## Abstract

**Background:**

Internationally, there is concern about the increased prescribing of pharmaceutical opioids for chronic non-cancer pain (CNCP). In part, this is related to limited knowledge about the long-term benefits and outcomes of opioid use for CNCP. There has also been increased injection of some pharmaceutical opioids by people who inject drugs, and for some patients, the development of problematic and/or dependent use. To date, much of the research on the use of pharmaceutical opioids among people with CNCP, have been clinical trials that have excluded patients with complex needs, and have been of limited duration (i.e. fewer than 12 weeks). The Pain and Opioids In Treatment (POINT) study is unique study that aims to: 1) examine patterns of opioid use in a cohort of patients prescribed opioids for CNCP; 2) examine demographic and clinical predictors of adverse events, including opioid abuse or dependence, medication diversion, other drug use, and overdose; and 3) identify factors predicting poor pain relief and other outcomes.

**Methods/Design:**

The POINT cohort comprises around 1,500 people across Australia prescribed pharmaceutical opioids for CNCP. Participants will be followed-up at four time points over a two year period. POINT will collect information on demographics, physical and medication use history, pain, mental health, drug and alcohol use, non-adherence, medication diversion, sleep, and quality of life. Data linkage will provide information on medications and services from Medicare (Australia’s national health care scheme). Data on those who receive opioid substitution therapy, and on mortality, will be linked.

**Discussion:**

This study will rigorously examine prescription opioid use among CNCP patients, and examine its relationship to important health outcomes. The extent to which opioids for chronic pain is associated with pain reduction, quality of life, mental and physical health, aberrant medication behavior and substance use disorders will be extensively examined. Improved understanding of the longer-term outcomes of chronic opioid therapy will direct community-based interventions and health policy in Australia and internationally. The results of this study will assist clinicians to better identify those patients who are at risk of adverse outcomes and who therefore require alternative treatment strategies.

## Background

Chronic non-cancer pain (CNCP) is a worldwide, common complaint. The prevalence of chronic pain (defined as pain present daily for three months or more) in the Australian population is 17% for males and 20% for females [1]. In one survey of 16 European countries, between 10% and 30% of participants reported chronic pain, 16% of whom said that some days the pain made them “want to die” [2]. Chronic pain can have a major impact on an individual and the community, with social, financial, employment and health costs [2].

CNCP is caused by many factors, including trauma. The varied aetiology probably impacts upon the effectiveness of treatment [1,3-5]. Physical and psychological factors such as depression and anxiety, a history of psychological trauma, and sleep problems moderate the pain experience [3]. Context is also important: relationships, occupational setting and culture all affect the experience and expression of pain [3]. Between 30-50% of those with chronic pain in the above study reported that their pain was not “adequately controlled” [2].

Effective behavioural treatments and non-opioid pharmacotherapy exist for CNCP [3], but even when a combination of interventions is used, some patients continue to suffer. The increase in the use of opioid analgesics for CNCP would suggest that there is conclusive evidence regarding their positive impact on pain reduction and quality of life. Indeed, qualitative and quantitative reviews of the evidence have concluded that chronic opioid therapy does produce clinically significant reductions in pain, albeit in the range of 2 to 3 points on a 0 to 10 visual analogue scale, or around 30% [6-8]. Nevertheless, reviewers have cautioned that patients need to be carefully selected and monitored, given that opioids are also associated with potentially serious harms and significant treatment drop out due to adverse effects [6,7]. Further, the degree to which pain reduction is achieved varies, and evidence on changes to quality of life and functional status is inconclusive [6]. Some of this variability in pain reduction may be related to the type of opioid used, with one meta-analysis finding that strong but not weak opioids reduced pain more effectively than other analgesics [9].

Controlled trials have evaluated pharmaceutical opioids in the treatment of a range of CNCP conditions and have demonstrated modest attenuation of pain [10]. Evidence indicates a modest short-term benefit; no studies have yet run for long enough to demonstrate long-term benefit of opioids for chronic non-cancer pain. The three systematic reviews of opioids for chronic non-cancer pain published to date provide evidence for a modest, short-term analgesic benefit [9,11,12]. This is a best-case scenario because RCTs select the sub-set of patients most likely to receive a clinical benefit and have short follow-up periods (average trial duration of five weeks), meaning they do not report on longer term outcomes [13,14]. An ongoing systematic review shows that the only evidence of long-term analgesic benefit (improved physical function and quality of life) is weak, because it is based on non-blinded studies with significant potential for reporting bias [6].

There is support from peak pain organisations of the use of opioids in the treatment of CNCP [2,15-17]. Debate continues about how, when, and in what manner opioids should be prescribed for this diverse patient group [3,18-22]. Consensus statements have recommended that prescription of opioids for chronic pain is considered only after following: a thorough assessment of the patient’s pain problem and history; development of a treatment plan; consultation with a pain specialist, if necessary; and regular reviews of patient progress [2,15].

Over the past decade, there has been increasing professional and public concern in a number of countries about pharmaceutical opioid use and related harms [23,24]. This has been driven by increases in prescribing of these drugs, especially in the USA and Canada. The increase in prescribing in Australia has been less than in Europe and the United States (US) [25-28], but nonetheless, between 1992–2007 the number of opioid prescriptions in Australia increased by around 300% [23]. This increase in prescribing has been accompanied by increased injection of some opioids by people who inject drugs [29]; increased concern about the appropriateness of prescribing these drugs for chronic non-cancer pain (CNCP) [6]; and for some patients, the development of problematic and/or dependent use.

The use of opioids, within, and outside the bounds of a doctor’s prescription has been cause for concern because of the risk of iatrogenic dependence [30], and opioid overdoses, with pharmaceutical opioids now comprising the majority of fatal and non-fatal drug overdoses in the US [24,31]. A review of 67 studies found that 11.5% of chronic pain patients engaged in aberrant drug-related behaviours such as diversion, prescription forgery, injecting, multiple episodes of prescription loss, escalating doses, and doctor shopping, 3.3% developed opioid abuse/dependence [32].

Despite this increasing concern, little is known about the magnitude of risk of such adverse events in patients prescribed opioids. Clinical trials, because they often exclude more complex patients, including those with comorbid conditions that may increase risks for developing opioid related problems (e.g. history of substance use disorder), typically find far lower rates of aberrant drug-related behaviours and abuse/dependence [32]. Many studies have also been of limited duration (~12 weeks) and few have examined aberrant drug use behaviours. Those of longer duration have had a small number of participants and therefore lack statistical power. Other studies have examined treatment of localised conditions, e.g. low back pain, or have evaluated a single drug or formulation of a drug. Only a few of the studies reviewed were prospective or longitudinal, thus limiting the conclusions that can be drawn. Further, there is little consensus regarding the diagnosis of opioid dependence in the context of chronic opioid treatment for chronic pain [33].

Little is known about the patterns of opioid prescribing for individual patients, and the long term outcomes for these patients. Small retrospective cohort studies conducted elsewhere have examined treatment duration, side effects, pain reduction, and adverse events [34] and aberrant behaviours [35]. Larger retrospective cohort studies have examined the risk of overdose [36], the impact on disability [37], non-medical use [38], conditions treated in older adults [39], and rates of adverse events [40].

The Pain and Opioid IN Treatment (POINT) study is an Australian-first study that aims to document patterns of pharmaceutical opioid prescribing, and risk of adverse events, in a prospective cohort of patients prescribed opioids for chronic non-cancer pain.

## Methods/Design

### Study aims

1. To examine patterns and outcomes of opioid analgesic use in a cohort of patients prescribed opioids for chronic non-cancer pain (CNCP).

2. To examine the demographic and clinical predictors of adverse events among a cohort of CNCP patients, including opioid abuse or dependence, medication diversion, other drug use, and overdose.

3. To identify factors which predict poor self-reported pain relief and other clinical outcomes.

### Study design and setting

The POINT study is a prospective cohort study of 1,500 persons who have been prescribed opioids for chronic non-cancer pain that follows their progress over two years and examine the predictors of clinical outcomes over this period. Participants provided consent to link data on health service utilisation and mortality data; the study will utilise data linkage to examine other clinical outcomes in the longer-term, such as opioid substitution therapy utilisation and hospital admissions. Prospective cohort studies can provide highly reliable and detailed data about a range of outcomes with the advantage of collecting data at the time or close to the time an event occurs, reducing the effects of recall bias [41] and making it easier to draw causal inferences about associations with later outcomes.

### Ethics

The study was approved by the Human Research Ethics Committee of the University of New South Wales (HREC reference: # HC12149). The study also received A1 National Pharmacy Guild Approval to approach pharmacists to assist with recruitment of participants (Approval n. 815). Approval was obtained by the Strategic Information Design and Governance Branch of the Department of Human Services to access Medicare data of participants that consent to access of their records (reference number: 2012/C011091).

### Sample size calculations

Power analyses were conducted to estimate minimum sample size required to assess the potential effects of opioid use on key clinical outcomes. We examine, dropout due to adverse events as an indicative example.

Power analyses were conducted using GLIMMPSE [42] to determine the minimum sample size to examine the potential effect of opioid use on key outcomes. As a conservative indicative example, we consider changes in pain score. Data on Australian prescriptions suggests that approximately 10% of opioid analgesic scripts can be defined as high dose [43]; we therefore assumed that at least 10% of the POINT cohort will be on a high dose of opioid analgesics. The variability of changes in pain score from baseline over a six month period was estimated from a recent Cochrane review [6]. Even with a drop-out rate as high as 25%, with an initial sample of 1500 we will be able to detect differences between low and high dose groups over time as small in magnitude as 0.30 at above 80% power and a Type I error rate of 5%. These calculations consider the effect of repeated observations over time (base correlation of 0.5 between adjacent repeated measures, with a linear exponential autoregressive decay rate of 0.05) and controlling for a normally distributed baseline covariate explaining up to 20% of the variation in pain scores. Given that other research questions relate to more equally balanced subgroups (e.g. comparisons of chronic neck/back pain vs. other types of pain which are distributed in a ratio of 4:1) or outcomes with less variability (e.g. changes in quality of life: [42]) then we can be confident that the POINT cohort is sufficiently powered to address the primary outcomes.

### Eligibility criteria

POINT participants were: 18 years or older; competent in English; and mentally and physically able to complete telephone and self-complete interviews; without memory or comprehension difficulties; living with chronic non-cancer pain (defined as pain present daily for a period of three months or more); prescribed a Schedule 8 opioid (an Australian classification of drugs of dependence that are subject to additional regulatory controls regarding their manufacture, supply, distribution, possession and use [44]); and had taken such opioids for CNCP for more than 6 weeks.

A history of injecting drug use (IDU) was not an exclusion criterion, but those currently prescribed pharmaceutical opioids for opioid substitution therapy (OST) for heroin dependence were not eligible. Nor were those taking opioids for cancer pain.

### Recruitment

A database of pharmacies and chemists across Australia and their contact details was purchased in May 2012 [45]. The list included 7,136 pharmacies. After removing duplicates, those that had closed down, or were not suitable for the study (i.e. located in a hospital or were a compounding pharmacy), we had a final list of 5,994 pharmacies.

Each fortnight, approximately, 100–150 pharmacies were randomly allocated into a Wave (with the exception of Tasmania which was sampled first). A flyer inviting to participate in research was faxed to all pharmacies in the Wave that had a fax number. Any pharmacies who expressed a lack of interest were not contacted further. Those who indicated they were interested in more information, or who did not respond to the fax, were called and the study was explained to a pharmacist who was asked if they were willing to participate.

Interested pharmacists were enrolled in the study for a 6-week period because it was expected that most people on prescription opioids would renew their script within this time. Pharmacists were asked to approach any customers that were prescribed a Schedule 8 opioid for CNCP for a period of greater than 6 weeks. We were confident that pharmacists were able to identify customers who had CNCP, rather than chronic cancer pain, by examining the customer’s record for other prescribed medications. Pharmacists were also able to use the medication history to confirm that customers had been on a prescription opioid for more than 6 weeks.

Customers who fit the above criteria were given a flyer about the study by the pharmacist. Interested customers were asked whether they would like the pharmacists to send their details to researchers, or if they wanted to contact researchers themselves. All flyers had a unique pharmacy number and pharmacists were reimbursed $20 for each eligible participant they referred into the study. POINT staff made reminder calls every fortnight for the 6 week period to pharmacists.

POINT staff determined the eligibility of those who were referred to the study, or who contacted the POINT team. Eligible participants went through a voluntary informed consent process. Those who were willing to participate, after being given details of the study, were booked in for their initial interview which was conducted over the phone and took approximately 1–1.5 hours. Detailed locator information was collected at Baseline to prevent sample attrition and this was updated at all follow-up assessments.

### Interview procedure

This study had four assessment waves: Baseline, T2 follow up (3 months), T3 follow up (12 months), and T4 follow up (24 months) (see Table 1).

**Table 1:** Time-points and data collection method of the POINT prospective cohort

**Baseline**	**T2 3 months**	**T3 12 months**	**T4 24 months**
• Phone interview	• Self-complete questionnaire (paper and pencil and online versions)	• Self-complete questionnaire (paper and pencil and online versions)	• Phone interview
• Self-complete questionnaire (paper and pencil and online versions)			• Self-complete questionnaire (paper and pencil and online versions)

Phone interviews were conducted by trained interviewers at the baseline and 24-month time-point. Interviewers had suicide assistance training, a minimum 3-year health or psychology degree, and were provided glossaries of general and chronic pain medications and conditions (see Additional files 1, 2 and 3). A self-completion survey was sent to all participants at each time-point. Participants were able to nominate to complete the self-complete survey either, online, by pen and paper, or on the telephone with a POINT team member. Participants were reimbursed $40 for the baseline interview, $25 at each 3-month and the 12-month time point and $60 at the final time point.

### Cohort maintenance strategies

There were a number of methods used to prevent attrition in the current study. Firstly, interested participants (pharmacists or participants) were called within a week from their expression of interest in the study. Secondly, participants were offered a variety of study completion methods, i.e. telephone, pen and paper and online. In addition to this, we offered to record verbal consent to participants who found it difficult to return the consent forms via mail. A variety of methods of study completion that are flexible and accommodating have been found to increase retention [46]. Thirdly, a detailed locator form was completed at the initial contact with participants. This form gathered detailed information on contact details of the participants, as well as the contact details of two secondary contacts (i.e. family, friends, medical professionals etc.). Finally, participants and pharmacists were reimbursed within a timely manner for their time and contribution to the research. Other methods to improve retention were letters thanking the participant for involvement in the study; this was sent after a payment had been made. A newsletter was sent annually to participants to provide study updates and progress.

### Project governance

A reference group was established for the study which included general practitioners, consumers, pain specialists, addiction medicine specialists, pharmacists and researchers. This reference group was consulted at the beginning of the study to assist in study and questionnaire design. They were also consulted throughout the study.

### Measures

Table 2 shows the measures, tools, domains and time-points at which data are collected. These measures were based on recommendations made under the auspices of the Initiative on Methods, Measurement, and Pain Assessment in Clinical Trials (IMMPACT). This Initiative involved 27 specialists from academia, governmental agencies, and the pharmaceutical industry who participated in a consensus meeting and identified core outcome domains and measures that should be considered in clinical trials of treatments for chronic pain [47,48]. The draft content of our interview was also reviewed and discussed by the POINT advisory committee.

**Table 2:** Measures, tools, domains and time-points for data collection for the POINT study

**Domain**	**Measure**	**Baseline**	**T1**	**T2**	**T3**	**Those who discontinue opioids**
**Demographics**						
Age and sex		✓	✕	✕	✕	✕
Marital status, employment, income		✓	✓	✓	✓	✓
Educational attainment		✓	✕	✕	✕	✓
**Pain**						
Pain	Brief pain Inventory (BPI) [49]	✓	✓	✓	✓	✓
Current chronic pain diagnosis, incident pain		✓	✓	✓	✓	✕
Illness and disability history	Chronic Conditions section of the CIDI 3.0 [50,51]	✓	✓	✓	✓	✕
Current physical disabilities	6-items from the Washington Group [52]	✓	✓	✓	✓	✓
**Physical functioning**						
Physical functioning	Brief Pain Inventory (BPI) [51], Short-Form (SF)-12 [53]	✓	✓	✓	✓	✓
Exercise	Exercise routine and how pain effects this	✓	✓	✓	✓	✓
Falls	Un-standardised questions examining falls	✓	✕	✓	✓	✓
Sleep	Medical Outcome Sleep scale (MOS) [54]	✓	✓	✓	✓	✓
Coping and pain	Pain: Self-Efficacy Questionnaire (PSEQ) [55,56]	✓	✓	✓	✓	✓
**Treatment**						
Past week use of prescription and OTC medications and dose	Self-complete 7 day medication diary	✓	✓	✓	✓	✓
Current prescribed medications and days of use in last month		✓	✓	✓	✓	✓
Other treatments for chronic pain		✓	✓	✓	✓	✓
Perceived effect of treatment	Patients’ Global Impression of Change scale (PGIC) [57]	✓	✓	✓	✓	✕
Beliefs about medicines	Beliefs about Medicines Questionnaires [58]	✓	✕	✓	✓	✓
Convenience of accessing medications		✓	✕	✕	✕	✕
Side-effects of opioid medication	Pain Assessment and Documentation Tool (PADT) [59]	✓	✓	✓	✓	✕
Barriers to treatment		✓	✓	✓	✓	✓
Reasons for discontinuance of opioids		✕	✕	✕	✕	✓
Aberrant opioid medication-related behaviours	Opioid Related Behaviours In Treatment ORBIT scale [60]	✓	✓	✓	✓	✕
Opioid Difficulties	Prescribed Opioid Difficulties Scale (PODS) [61]	✓	✕	✓	✓	✓
**Quality of life**	WHO-QoL-BRIEF [62]	✓	✓	✓	✓	✓
**Mental health**						
Mental health history		✓	✕	✕	✕	✕
Depression	Patient Health Questionnaire −9 (PHQ-9) [63,64]	✓	✓	✓	✓	✓
General Anxiety disorder	Patient Health Questionnaire (GAD) [65]	✓	✓	✓	✓	✓
Social Anxiety	Social Interaction Anxiety Scale (SIAS) [66]	✓	✓	✓	✓	✓
Social Phobia	Social Phobia Scale (SPS) [66]	✓	✓	✓	✓	✓
Agoraphobia	From the MINI International Neuropsychiatric Interview [67]	✓	✓	✓	✓	✓
Post-Traumatic Stress Disorder	PC-PTSD [68]	✓	✓	✓	✓	✓
Borderline personality disorder screener	Screener from the CIDI 3.0 [50]	✓	✕	✕	✕	✕
Child abuse	Childhood Trauma Questionnaire [69]	✓	✕	✕	✕	✕
Suicidality and self-harm	National Survey of Mental Health and Well-Being	✓	✕	✓	✓	✓
Social support	(SPQ), MOS Social Support [70]	✓	✓	✓	✓	✓
Locus of control	Multidimensional Health Locus of Control (B, C) [71]	✓	✕	✓	✓	✓
**Substance use**						
Lifetime and current drug use, illicit drug market		✓	✓	✓	✓	✓
Drug and alcohol abuse and dependence	CIDI 3.0 [50]	✓	✕	✕	✕	✓

### Data linkage

Consent was also obtained from POINT participants to link their data with the following datasets:

#### Medicare

Medicare (the Australian national health care scheme) collects data on all patient services with medical practitioners in Australia. The following data fields are collected: item number; Medicare benefit; date of service, processing or referral; indication of whether or not the service was provided in hospital; number of services, rendered or referred; and State of patient.

#### National Death Index (NDI)

The NDI is a fully identified database held by the Australian Institute of Health and Welfare (AIHW) and contains mortality data collected from each of the State and Territory Births, Deaths and Marriage Registers. It collects information on deaths and includes date, state, and causes (primary causes for all records, secondary causes for deaths in 1997 and later).

#### Opioid substitution treatment

The Pharmaceutical Drugs of Addiction System (PHDAS) database is a comprehensive record of all individuals in NSW who received addictive drugs dispensed by clinicians with the authorisation of the NSW Director-General of Health since 1985. The PHDAS is a fully identified database of all methadone and buprenorphine recipients (i.e. first name, last name, date of birth, sex, postcode of residence). As proof of identity must be shown to the prescribing doctor, the name and date of birth variables are of high quality in this dataset. The database also records patient admissions and exits from the treatment program, and the type of pharmacotherapy dispensed.

The data linkage with PHDAS will be performed by probabilistic record linkage software by staff at Medicare (PBS and Medicare data), AIHW (NDI data) and NSW Health (PHDAS, NSW hospital data). Variables used for matching purposes will include: full name, date of birth, sex, date and state of last known contact where available. Matching will be performed in several stages (“passes”), commencing with the strictest of criteria (e.g.: an exact match of full name and date of birth) to more relaxed criteria (e.g.: slight spelling and date variations permitted). The records matched in each pass will be weighted according to the quality of the match.

### Data analysis

Descriptive statistics will be used to determine the quantity and type of opioid and other medication use, and the prevalence of other key variables such as psychiatric disorders, demographics and physical health problems. A range of standard statistical techniques will be used depending upon the research question, such as logistic and linear regressions and survival analysis. Latent class analysis may be used to examine whether there are differing latent classes of CNCP participants, and whether such classes predict clinical outcomes. Generalised estimating equations (GEE), (statistical techniques for analysing correlated data) will be used to examine whether the quantity and type of opioid analgesic use predicts later health outcomes. Latent growth curve modeling (LGCM) within a structural equation modeling framework will examine whether the quantity and type of opioid use predicts outcomes such as pain relief, quality of life, functional status, side effects, and psychiatric disorders. All GEE and LGCM analyses will control for a range of confounding factors.

## Discussion

This project is the first large-scale Australian cohort study that will rigorously examine opioid analgesic use among chronic pain patients, and examine the relationship of opioid use to important health outcomes including mortality. This study will be the first to comprehensively examine the extent to which opioid therapy for chronic pain is associated with pain reduction, changed quality of life, mental and physical health outcomes, aberrant medication behavior and substance use disorders.

The study will shed light on the extent to which patients experience problematic opioid use, some of the precursors and protective factors to problematic use, and the consequences of problematic opioid use related to chronic opioid therapy. It will lead to improved knowledge of dose changes over time, and the positive and negative outcomes associated with changes in patterns of opioid use over time.

Improved understanding of the longer-term outcomes of chronic opioid therapy will direct community-based interventions and health policy in Australia. Regulators across jurisdictions currently use different criteria for authorising long-term opioid therapy, and different criteria for identifying at-risk patients. The results of this study will assist doctors and regulators in Australia to better identify those patients who are at risk of adverse outcomes and who therefore require alternative treatment strategies.

Finally, the project will achieve the establishment of a cohort of Australians with chronic pain. The project will provide the groundwork for further follow-up of the sample to determine the longer-term outcomes for chronic pain patients.

## Competing interests

BL, LD, RPM and NL have received untied educational grants from Reckitt Benckiser for the post-marketing surveillance of opioid substitution therapy medications in Australia, and the development of an opioid-related behavior scale. SN has also received untied educational grants from Reckitt Benckiser, as has NL for research examining buprenorphine-naloxone Film. LD, BL, MF, NL, and RB have received untied educational grants from Mundi Pharma to conduct surveillance of the use of pharmaceutical opioids in Australia. All such studies’ design, conduct and interpretation of findings are the work of the investigators; the funders had no role in those studies.

## Authors’ contributions

LD, RB, NL, MF, MC, WH, RPM, SN and FS contributed to the development of the study for the purposes of the funding proposal and development of the study design. GC, LD, BL, SN and BH led writing for the first draft. CK drafted the content for the appendices. All authors contributed to the critical review of the manuscript. All authors read and approved the final manuscript.

## Pre-publication history

The pre-publication history for this paper can be accessed here:

http://www.biomedcentral.com/2050-6511/15/17/prepub

## Supplementary Material

Additional file 1 Glossary of conditions that may lead to chronic pain. Click here for file

Additional file 2 General pain categories. Click here for file

Additional file 3 Medications that may be used for pain conditions. Click here for file
