# Supplementary material for: Cohort protocol paper: The Pain and Opioids In Treatment (POINT) study
Source: BMC Pharmacol Toxicol. 2014 Mar 20;15:17. doi: 10.1186/2050-6511-15-17 (PMC4000138; doi:10.1186/2050-6511-15-17)
Supplement: Additional file 1 — Glossary of conditions that may lead to chronic pain. [file 2050-6511-15-17-S1.docx]

**Additional file 1 Glossary of conditions that may lead to chronic pain**

| **CONDITION** | **PAIN LOCATION** | **DESCRIPTION** | **TYPE OF PAIN** |
| --- | --- | --- | --- |
| Achalasia | Throat/Chest Pain | The esophagus has problems moving properly, causing difficulty swallowing, regurgitation, and pain. No known cause; can be treated but not cured (Park W, 2005) (Lake & Wong, 2006)**.** | Visceral |
| Alcohol dependence | Peripheral Pain | Alcohol dependence can contribute to peripheral neuropathy - pain in extremities such as hands and feet (Muller, Koch, Von Specht, Volker, & Munch, 1985). | Neuropathic |
| Ankylosing spondylitis (aka Bechterew’s disease, Bechterew syndrome and Marie Struempell disease) | Back/Joint Pain | Arthritis of the spine. It causes swelling between your vertebrae, which are the disks that make up your spine, and in the joints between your spine and pelvis. Ankylosing spondylitis is an autoimmune disease. This means your immune system, which normally protects your body from infection, attacks your body's own tissues. The disease is more common and more severe in men. It often runs in families (American Chronic Pain Association, 2012) (London Pain Clinic, 2006) . | Deep Somatic |
| Arachnoiditis | Back Pain | A pain disorder caused by the inflammation of the arachnoid, one of the membranes that surround and protect the nerves of the spinal cord.  Inflammation can sometimes lead to the formation of scar tissue and adhesions, which cause the spinal nerves to “stick” together. If arachnoiditis begins to interfere with the function of one or more of these nerves, it can cause a number of symptoms, including numbness, tingling, and a characteristic stinging and burning pain in the lower back or legs (American Chronic Pain Association, 2012). | Neuropathic |
| Bursitis | Joint Pain | Bursitis is a painful condition that affects the small fluid-filled pads — called bursae — that act as cushions among your bones and the tendons and muscles near your joints. Bursitis occurs when a bursa becomes inflamed. The most common locations for bursitis are in the shoulders, elbows or hips. Bursitis often occurs in joints that perform frequent repetitive motion (American Chronic Pain Association, 2012). | Deep Somatic |
| Carpal Tunnel Syndrome | Peripheral Pain | Swelling in the wrist tunnel irritates the median nerve. CTS causes tingling, numbness and pain over the thumb, first and middle fingers (Jacques, 2009). | Neuropathic |
| Central Pain Syndrome | Generalised Pain | Neurological condition caused by damage to or dysfunction of the central nervous system (CNS), which includes the brain, brainstem, and spinal cord. This syndrome can be caused by: stroke, multiple sclerosis, tumors, epilepsy, brain or spinal cord trauma, or Parkinson's disease. Central pain syndrome may affect a large portion of the body or may be more restricted to specific areas, such as hands or feet. Pain is typically constant, may be moderate to severe in intensity, and is often made worse by touch, movement, emotions, and temperature changes. There are several common pain sensations: burning, "pins and needles;" pressing, lacerating, or aching pain; and brief, intolerable bursts of sharp pain akin to a dental probe on an exposed nerve. Central pain syndrome often begins shortly after a causative injury/damage, but may be delayed by months/years (American Chronic Pain Association, 2012) | Neuropathic |
| Cervical Dystonia/Spasmodic Torticollis | Neck Pain | Cervical dystonia, also called spasmodic torticollis, is a painful condition in which your neck muscles contract involuntarily, causing your head to twist or turn to one side. A rare disorder that can occur at any age, cervical dystonia most often occurs in middle-aged people (Jankovic J. , 2006) | Deep somatic |
| Chronic Fatigue Syndrome | Generalised Pain | An illness without a known cause that is characterized by long-term exhaustion, muscle weakness, depression, pain and sleep disturbance; widespread muscle and joint pain, along with frequent headaches, are commonly reported (Blatman Pain Clinic). | Deep Somatic |
| Chronic Pancreatis | Abdominal/Pelvic Pain | Continuing, chronic, inflammatation of the pancreas, leading to chronic abdominal pain and/or impairment of endocrine and exocrine function of the pancreas. Chronic pancreatitis is a completely different inflammation process from acute pancreatitis. acute pancreatis is fully curable but chronic pancreatitis is a chronic, irreversible inflammation leading to fibrosis with calcification (American Chronic Pain Association, 2012). | Visceral |
| Cluster Headaches | Headaches | Often confused with migraines, these severe headaches are usually caused by enlarged blood vessels leading into the head (Blatman Pain Clinic). | Deep somatic |
| Complex Regional Pain Syndrome | Generalised Pain | A chronic neurological syndrome characterized by: severe burning pain; pathological changes in bone and skin; excessive sweating; tissue swelling; extreme sensitivity to light & touch (American Chronic Pain Association, 2012). | Neuropathic |
| Compression Fractures | Back Pain | Commonly associated with osteoporosis, compression fractures occur when brittle vertebral bones collapse (Jacques, 2009). | Deep somatic |
| Crohn’s Disease | Abdominal/Pelvic Pain | Inflammation of the digestive system, causing abdominal/pelvic pain. It is one of a group of diseases called inflammatory bowel disease (American Chronic Pain Association, 2012) | Visceral |
| Cystic Fibrosis | Generalised Pain | Cystic Fibrosis (CF) is a genetic disorder that affects the lungs and other organs; it is characterised by scarring and cyst formations in the pancreas. Pain is a common symptom; about 60% of adults with Cystic Fibrosis report negative effects of pain on their daily life. Recurrent lung and sinus infections, surgery, pneumothorax, and intestinal secretions can lead to significant pain.  Bone and joint pain is associated with osteomalacia and osteoporosis which result from malabsorption of essential minerals and vitamins (American Chronic Pain Association, 2012) (Broome & Subramaniam, 2003) (Filippo Festini, 2004). | Combination |
| Dercum’s Disease | Generalised Pain | Extremely rare disorder characterized by multiple, severely painful growths consisting of fatty tissue (lipomas). These growths mainly occur on the trunk, the upper arms and upper legs and are found just below the skin. Pain may be caused by these growths pressing on nearby nerves.The exact cause of Dercum's disease is unknown (American Chronic Pain Association, 2012). | Superficial Somatic |
| Diabetes (Diabetic Neuropathy) | Peripheral Pain | Sensory nerve damage is a common side effect of diabetes. It can cause numbness or pain, most often in the hands or feet (Jacques, 2009). | Neuropathic |
| Dystonia | Generalised Pain | A disorder where muscles involuntarily contract, causing an uncontrollable and often painful twisting and cramping of the affected body part. Symptoms can be mild or severe and can affect the entire body or one area. Most cases of dystonia, however, tend to affect only one body part — often the neck, the face or an arm (American Chronic Pain Association, 2012) (Jankovic J. , 2006). | Deep Somatic |
| Endrometriosis | Abdominal/Pelvic Pain | Tissue which lines the uterus (tissue called the endometrium) is found outside the uterus. This misplaced tissue develops into growths or lesions and may cause scarring. Less commonly they are found in the lung, arm, thigh, and other locations. Pelvic pain is a common symptom (Office of Communications and Public Liaison, 2012). | Visceral |
| Erythromelalgia (Mitchell's Disease) | Peripheral Pain | Episodes of pain, redness, and swelling in various parts of the body, particularly the hands and feet. These episodes are usually triggered by increased body temperature. The pain can be so debilitating that it impedes everyday activities such as wearing shoes and walking (American Chronic Pain Association, 2012). | Neuropathic |
| Eye Strain Headaches | Headaches | Ocular muscles become fatigued and cause head pain. This is usually caused by sitting at a computer for too long, or wearing the wrong eyeglass prescription (Jacques, 2009). | Deep somatic |
| Failed Back Surgery syndrome (FBSS) | Back Pain | A very generalized, non-medical term that is often used to describe the chronic pain condition of patients who have not had a successful result with back surgery or spine surgery (American Chronic Pain Association, 2012). | Combination |
| Fibromyalgia | Generalised Pain | Widespread muscle fatigue and pain, often accompanied by chronic fatigue, sleep disorders and irritable bowel syndrome. Fibromyalgia is medically unexplained but thought to be neuropathic in nature (Jacques, 2009). | Neuropathic |
| Frozen Shoulder (aka adhesive capsulitis) | Joint Pain | The connective tissue surrounding the shoulder joint becomes inflamed, causing chronic pain. Pain is usually constant, worse at night, and when the weather is colder. The exact cause is unknown, and the condition can last for years (American Academy of Orthopedic Surgeons, 2011) (Ewald, 2011)**.** | Deep Somatic |
| Gout | Joint Pain | Caused by deposition of uric acid crystals in the joint (usually in the foot), causing inflammation and pain (American Chronic Pain Association, 2012). | Deep Somatic |
| Hidradenitis suppurativa (fox-den disease) | Generalised Pain | Chronic skin disease that results in extremely painful open wounds that do not heal. Treatment is difficult (New Zealand Dermatological Society Inc, 2012). | Superficial Somatic |
| Inflammatory Bowel Disease | Abdominal/Pelvic Pain | A chronic condition where the gastrointestinal tract, intestine or colon becomes inflammed, resulting in abdominal pain and cramping (see also Crohn's disease) (Office of Communications and Public Liaison, 2012). | Visceral |
| Interstitial Cystisis (Painful bladder syndrome) | Abdominal/Pelvic Pain | A chronic condition characterized by a combination of uncomfortable bladder pressure, bladder pain and sometimes pain in the pelvis, which can range from mild burning or discomfort to severe pain. The severity of symptoms caused by interstitial cystitis often fluctuates, and some people may experience periods of remission (Office of Communications and Public Liaison, 2012) (American Chronic Pain Association, 2012). | Visceral |
| Irritable Bowel Syndrome | Abdominal/Pelvic Pain | IBS affects the colon, or large bowel. IBS is not a disease; it's a functional disorder, meaning that the bowel doesn't function correctly. The most common symptoms of IBS are abdominal pain or discomfort often reported as cramping, bloating, gas, diarrhoea, and/or constipation (American Chronic Pain Association, 2012). | Visceral |
| Knee Injury | Knee Pain | Knee Injury: Your knee joint is made up of bone, cartilage, ligaments and fluid. Muscles and tendons help the knee joint move. When any of these structures is hurt or diseased, you have knee problems. Knee problems can cause pain and difficulty walking (American Chronic Pain Association, 2012). | Deep Somatic |
| Loin Pain-Haematuria Syndrome (also known as LPHS) | Abdominal/Pelvic Pain | This syndrome is a combination of kidney pain and blood in the urine. The pain may be a continuous dull ache, or intermittent, coming on only occasionally (American Chronic Pain Association, 2012). | Visceral |
| Lupus | Generalised Pain | A chronic, autoimmune disease that can damage any part of the body (skin, joints, and/or organs inside the body). The immune system malfunctions in lupus, creating antibodies that attack and destroy healthy tissue. These antibodies cause inflammation, pain, and damage in various parts of the body (American Chronic Pain Association, 2012). | Combination |
| Meralgia Paresthetica (Bernhardt-Roth Syndrome) | Peripheral Pain | A condition characterized by tingling, numbness and burning pain in the outer part of the thigh, caused by either compression of the nerve that supplies sensation to the skin surface of the thigh or damage from a condition like diabetes or an injury (American Chronic Pain Association, 2012). | Neuropathic |
| Migraines | Headaches | Migraines can be caused by nervous system triggers or hormonal changes in the body. They often cause pain on one side of the head or face, and may be accompanied by nausea and sensitivities to light, sounds or smells (Jacques, 2009) (Blatman Pain Clinic). | Deep somatic |
| Multiple Sclerosis | Generalised Pain | Multiple Sclerosis (MS) is an autoimmune disease that affects the central nervous system, attacking and damaging the nerves in the body. Pain is a common symptom in those with Multiple Sclerosis. Chronic pain is experienced by 64-69% of people with MS (Jacques, 2009) (Office of Communications and Public Liaison, 2012) (MS Australia, 2009). | Neuropathic |
| Muscle Tension Headache | Headaches | Often caused by stress, fatigue or “sleeping wrong,” muscles of the neck, shoulders and scalp tighten. This causes pressure on the head, leading to pain (Jacques, 2009). | Deep somatic |
| Myofascial Pain Syndromes | Generalised Pain | Myofascial pain syndromes cause weakness, restriction of motion, acute pain, and chronic pain; it comes from myofascial trigger points that form in muscle tissue and connective tissue. More active trigger points are tender and generate referral pain patterns when they are pushed on. They cause varying types of pain that include cramping, burning, aching, numbness and tingling. Less active myofascial trigger points can predispose a person to acute pain attacks and cause: muscle weakness, early fatigue and restriction of motion, with or without pain (i.e. frozen shoulder, tight hamstrings) (Blatman Pain Clinic). | Deep somatic |
| Occipital Neuralgia | Headaches | A distinct type of headache characterized by piercing, throbbing, or electric-shock-like chronic pain in the upper neck, back of the head, and behind the ears, usually on one side of the head.  Typically, the pain of occipital neuralgia begins in the neck and then spreads upwards.  Some individuals will also experience pain in the scalp, forehead, and behind the eyes.  Their scalp may also be tender to the touch, and their eyes especially sensitive to light (American Chronic Pain Association, 2012). | Neuropathic |
| Osteoarthritis | Joint Pain | Wear and tear on joints over time. It is common in the elderly, and usually affects one or more of the larger joints in the body (Jacques, 2009). | Deep somatic |
| Osteoporosis | Generalised Pain | Osteoporosis is a disease characterized by low bone mass and deterioration of the bone tissue. This process of bone thinning leads to an increase in bone fragility and the consequent risk of fracture. The hip, spine and wrists are the most common areas of osteoporosis-related bone fractures, though these can actually occur almost anywhere in the skeleton. Osteoporosis runs a major risk of passing by undiagnosed as it can progress painlessly until a bone actually breaks. In fact, due to this, osteoporosis is often referred to as the ‘silent thief’, lacking any obvious signs of onset. Broken bones can cause chronic pain While a hip fracture usually requires hospitalization and major surgery, spinal or vertebral fractures also have serious consequences such as loss of height, severe back pain and deformity (Office of Communications and Public Liaison, 2012). | Combination |
| Ovarian cysts | Abdominal/Pelvic Pain | Fluid-filled growths on the ovaries. They can recur and cause chronic pelvic pain (Medicine Online, 2004). | Visceral |
| Paget’s disease of bone | Generalised Pain | Bones grow larger and weaker than normal, causing deep bone pain. They also might break easily. The disease can lead to other health problems, too, such as arthritis and hearing loss. The disease is most common in the spine, pelvis, skull and legs. The disease might affect one or several bones. It is most common in older people (American Chronic Pain Association, 2012). | Deep Somatic |
| Parkinson’s disease | Generalised Pain | Degenerative disorder of the central nervous system. Joint pain is commonly associated with this disease; the disease can also impair the senses, including an increased or decreased sensation of pain (Jankovic J. , 2008). | Combination |
| Phantom Limb Pain | Peripheral Pain | Pain that is felt in an amputated limb. . Phantom limb pain can be mild to extremely painful. In some cases, phantom limb pain can be disabling and can lead to a lifelong struggle with chronic pain. When phantom limb pain continues for more than six months, the prognosis for spontaneous improvement is poor (Blatman Pain Clinic) (American Chronic Pain Association, 2012). | Neuropathic |
| Polymyalgia rheumatica | Generalised Pain | An inflammatory disorder that causes muscle pain and stiffness, primarily in the neck, shoulders, upper arms, hips and thighs. Symptoms usually begin quickly over a few days. More common in older people. Polymyalgia rheumatica is related to and may coexist with another inflammatory disorder called giant cell arteritis, which can cause headaches, visual impairment, jaw pain and other symptoms (American Chronic Pain Association, 2012). | Deep Somatic |
| Post-Mastectomy Pain Syndrome | Peripheral Pain | A type of chronic postoperative pain that persists after a mastectomy or other type of breast surgery is performed. Post-mastectomy pain is often neuropathic; may be caused by damage to the nerves in the breast and underarm area, or the development of a neuroma (American Chronic Pain Association, 2012). | Neuropathic |
| Refractory angina or Intractable angina | Generalised Pain | Refractory angina or Intractable angina is a condition in which patients of heart disease continue to suffer from recurrent restricting angina, even though they are following the right medication plan. Refractory angina is a chronic and incapacitating condition and often responds poorly to treatment (American Chronic Pain Association, 2012). | Combination |
| Repetitive Strain Injury | Peripheral Pain | A painful condition affecting people who overuse muscles as a result of, for example, regularly operating a computer keyboard and mouse or playing the piano. It is usually caused by some combination of inflammation and myofascial trigger points in the painful areas (Blatman Pain Clinic). | Combination |
| Repetitive Strain Injury | Joint Pain | Common in athletes, frequent injuries over time can result in chronic pain. Typically these involve larger joints like the knee or the shoulder (Jacques, 2009). | Deep somatic |
| Restless Leg Syndrome | Peripheral Pain | A powerful urge to move the legs. Legs become uncomfortable when lying down or sitting. Some people describe it as a creeping, crawling, tingling or burning sensation. Moving relieves the discomfort, but not for long (American Chronic Pain Association, 2012). | Neuropathic |
| Rheumatoid Arthritis | Joint Pain | Often present in early adulthood, causes swelling in the joint spaces. Eventually it also damages bones, ligaments and tendons (Jacques, 2009). | Deep somatic |
| Sacroiliac Joint Dysfunction | Back Pain | Dysfunction in the sacroiliac joint, or SI joint, is thought to cause low back and/or leg pain. The leg pain can be particularly difficult, and may feel similar to sciatica or pain caused by a lumbar disc herniation (American Chronic Pain Association, 2012). | Combination |
| Sciatica | Back/Leg Pain | The sciatic nerve runs from your back to your feet. Compression or damage of this nerve can cause pain to shoot down the leg on one side of the body (Jacques, 2009). | Neuropathic |
| Shingles (Postherpetic Neuralgia) | Generalised Pain | A painful condition affecting your nerve fibers and skin; symptoms can become chronic. Postherpetic neuralgia is a complication of shingles. During an initial infection of chickenpox, some of the virus remains in your body, lying dormant inside nerve cells. Years later, the virus may reactivate, causing shingles (a viral infection of the nerve roots). In Postherpetic neuralgia, shingles damaged skin nerves, causing pain signals to be sent to the brain (American Chronic Pain Association, 2012) (Helgason, Petursson, Gudmundsson, & Sigurdsson, 2000). | Neuropathic |
| Slipped or bulging disc (herniated disc) | Back Pain | Often the result of twisting or lifting injuries. Damaged discs protrude into the spinal canal, pressing against nerves as they exit the spinal cord (Jacques, 2009). | Deep somatic |
| Soft Tissue Damage | Back Pain | Heavy lifting or trauma can cause damage to back muscles, ligaments and tendons (Jacques, 2009). | Deep somatic |
| Somatization disorder | Generalised Pain | Recurring, multiple, clinically significant complaints about pain in different body sites that are not fully explained by a known medical condition (Allen, Woolfolk, Escobar, Gara, & Hamer, 2006). | Psychogenic |
| Sphincter of Oddi dysfunction | Abdominal/Pelvic Pain | Sphincter of Oddi dysfunction refers to structural disorders where there is an obstruction of bile and pancreatic juice flow, causing pain (American Chronic Pain Association, 2012). | Visceral |
| Spinal Stenosis | Back Pain | Narrowing of the spinal canal, which can compress nerves (Blatman Pain Clinic). | Deep somatic/Neuropathic |
| Spinal stenosis | Back/Buttock | Spinal stenosis is a condition where the narrowing of the spinal canal can cause spinal cord or nerve pinching which leads to chronic pain in the buttocks, limping, lack of feeling in the lower extremities, and decreased physical activity (American Chronic Pain Association, 2012). | Neuropathic |
| Trigeminal Neuralgia (Fothergill’s disease) | Headaches | A neuropathic disorder of trigeminal nerve in the face. It causes episodes of intense pain in any or all of the following: the ear, eye, lips, nose, scalp, forehead, teeth or jaw on one side of the face. Can be caused by Multiple Sclerosis (American Chronic Pain Association, 2012) (Office of Communications and Public Liaison, 2012). | Neuropathic |
| Stroke (Post-Stroke Pain Syndrome) | Generalised Pain | Pain that follows a stroke (a rapid loss of brain function due to a disturbance in the brain's blood supply) is termed post-stroke pain. Most strokes do not cause pain, only numbness. However, sometimes people experience ongoing deep burning, pins and needles sensations and often muscle contractions in various parts of the body, thought to be caused by damage to the neurons in the brain (American Chronic Pain Association, 2012)**.** | Neuropathic |
| Structural Deformities | Back Pain | Spinal abnormalities such as scoliosis put strain on the muscles that control posture, causing pain and fatigue (Jacques, 2009). | Deep somatic |
| Syringomyelia | Generalised Pain | A rare disorder that causes a cyst to form in the spinal cord which gets bigger and destroys part of the spinal cord. Damage to the spinal cord from the syrinx can cause symptoms such as Pain and weakness in the back, shoulders, arms or legs,  headaches , inability to feel hot or cold (6). | Neuropathic |
| Temporomandibular joint dysfunction (3) | Headaches/Joint Pain | A painful condition involving the temporomandibular joint and the muscles used for chewing, sometimes causing clicking sounds and restricted jaw movement. It is a common and sometimes overlooked cause of chronic headaches (2). | Deep somatic |
| Thorocotomy (cut in the chest for surgery) | Generalised Pain | Post Thorocotomy Pain Syndrome Pain after thoracotomy (cutting the chest open for surgery) is very severe and chronic pain, probably the most severe pain experienced after surgery, most likely caused by trauma to the intercostal nerve during surgery (6) (Karmaker & Ho, 2004). | Combination - increasing evidence to show it is neuropathic |
| Transverse Myelitis | Generalised Pain | A neurological disorder caused by inflammation across both sides of one segment of the spinal cord. Attacks of inflammation can damage or destroy myelin, the fatty insulating substance that covers nerve cell fibers. This damage causes nervous system scars that interrupt communications between the nerves in the spinal cord and the rest of the body (6). | Neuropathic |
| Traumatic Brain Injury | Generalised Pain | A traumatic brain injury can cause damage to the way the nerves transmit signals to the brain; can be a cause of Central Pain Syndrome | Neuropathic |
| Traumatic Fracture | Back Pain | Falls from elevation, car accidents or crush injuries can cause painful vertebral fractures (1) | Deep somatic |
| Vulvodynia | Abdominal/Pelvic Pain | Chronic vulvar pain without an identifiable cause. The location, constancy and severity of the pain vary among sufferers. Some women experience pain in only one area of the vulva, while others experience pain in multiple areas. The most commonly reported symptom is burning, but women’s descriptions of the pain vary (6) (London Pain Clinic, 2006). | Visceral |
| Whiplash (Whiplash syndrome) | Generalised Pain | Whiplash a non-medical term. It is caused by the neck and head being thrown suddenly backward then forward upon impact. The impact forces the neck and head beyond their normal range of movement, causing tissue damage and pain (6). In some cases, patients of whiplash continue experiencing pain even after a whiplash trauma is over: headaches and pain, reduced movement at the back of the neck, tingling in arms, lumbar pains, fatigue, sleep disturbances and reduced libido. Up to 60 percent of all whiplash injury patients suffer from lower back pain afterwards. This is commonly caused due to the injury to the discs, facet joints of the low back or the sacroiliac joints (London Pain Clinic, 2006). | Combination |
