# Supplementary material for: Cohort protocol paper: The Pain and Opioids In Treatment (POINT) study
Source: BMC Pharmacol Toxicol. 2014 Mar 20;15:17. doi: 10.1186/2050-6511-15-17 (PMC4000138; doi:10.1186/2050-6511-15-17)
Supplement: Additional file 2 — General pain categories. [file 2050-6511-15-17-S2.docx]

**Additional file 2: General pain categories**

| **Category** | **Description** | **Common Medications** |
| --- | --- | --- |
| **Joint Pain (Arthritis/**  **Rheumatism)** | OsteoArthritis – inflammation of joints – osteoarthritis is commonly associated with older populations.  Rheumatoid Arthritis – has a younger age of onset than Osteoarthritis; the body’s immune system starts to attack tissues, including joint tissues, which causes inflammation and pain. | - Analgesics (pain relievers), from Paracetamol to opiates. - Non-steroidal anti-inflammatory drugs (NSAIDs) such as naproxen & ibuprofen (Nurofen), ketoprofen (Orudis) - Corticosteroids (taken by tablet or injection) (prednisolone, cortisone injection) - Disease-modifying anti-rheumatic drugs (DMARDS) – suppress the immune system (biological DMARDS block substances that cause inflammation). Halting the inflammation reduces pain (Orencia, Humira) (Arthritis Information Sheet: Medicines and arthritis, 2012) |
| **Chronic back or neck problems** | Commonly pain in the lower back but can be in the neck. | - Analgesics, including opioids - NSAIDs - Muscle Relaxants (benzodiazepines such as Valium or Xanax) - Antidepressants (Bogduk, 2004) |
| **Frequent or severe headaches** | Most common form of chronic headache pain is Migraine – chronic, moderate-severe headache & nausea.  Nerve Pain (Neuralgias) can also occur as a headache. | - NSAIDS, including Naproxen or ketorolac injections - Triptan (Seratonin antagonist) such as Sumatriptan - Antidepressants (Tricyclic or SSRI’s) may help prevent migraines - Corticosteroid injection - Ergot alkaloid - Ergotamine (vasoconstrictor alkaloid often combined with caffeine for treatment of migraines, i.e. Cafergot) - Combination of paracetamol, codeine and antihistamine doxylamine (Dolased or Mersyndol) (Duckro, 1999) (Consumer Medicine Information: Mersyndol, 2007) (Consumer Medicine Informaiton: Dolased Analgesic, 2004) |
| **Generalised Pain** | Common chronic generalised pain conditions (pain throughout the body) include:   - Complex Regional Pain Syndrome (CRPS) – intense, burning chronic pain caused by nerve damage  1. Reflex Sympathetic Dystrophy (CRPS1) – chronic nerve disorder occurs most often in arms or legs after a minor injury 2. Causalgia (CRPS2) - caused by injury to the nerve  - Fibromyalgia – chronic widespread pain, fatigue and heightened and painful response to pressure. - Chronic fatigue syndrome – chronic muscle pain, joint pain, or headaches are some of the symptoms of this disorder. | - Analgesics (including opioids) - NSAIDS - Antidepressants such as Duloxetine used for control of neuropathic pain conditions like Fibromyalgia. - Anticonvulsants such as Lyrica are also used for neuropathic pain conditions Fibromyalgia (Consumer Medicine Information: Lyrica, 2011) (Eustice, 2008) |
| **Visceral Pain** | Pain that is felt inside the body. Pain from the organs. Some common conditions involving visceral pain include:   - Endometriosis – a condition affecting women caused by an excess of cells in the uterine cavity. Major symptom of this condition is recurring pelvic pain - Chronic Pancreatis - inflammation of the pancreas | Opioids are a common treatment for visceral pain (Giamberardino, 2005). |
| **Other Neuropathic Pain Conditions** | Neuropathic pain is caused by damaged or malfunctioning nerves. It is often described as different from other forms of pain and is often described as: electric shocks, burning, or “heavy” sensations. It can occur by itself or along with other forms of pain in many conditions. Some examples include:   - Alcohol dependence and Diabetes can contribute to peripheral neuropathy (pain at extremities such as hands and feet) - Phantom pain – pain from a part of the body that has been lost, common experience of amputees and can become chronic. - Operations such as mastectomies or thorocotomy or amputations can leave ongoing chronic neuropathic pain at the affected site. | Neuropathic pain is difficult to treat compared to somatic pain. A combination of treatments can be used:   - Antidepressants of the Tricyclic type - Anticonvulsants (Gabapentin) - Analgesics like opioids (may be ineffective but pain that does not respond to other treatments may respond to opioids) - Local anaesthetics - Ketamine (Helme, 2006) |
