# Supplementary material for: Cohort protocol paper: The Pain and Opioids In Treatment (POINT) study
Source: BMC Pharmacol Toxicol. 2014 Mar 20;15:17. doi: 10.1186/2050-6511-15-17 (PMC4000138; doi:10.1186/2050-6511-15-17)
Supplement: Additional file 3 — Medications that may be used for pain conditions. [file 2050-6511-15-17-S3.docx]

**Additional file 3: Common medications used for pain conditions**

| **Brand of Medication** | **Name of Drug** | **Classification** | **Type** | **Comment** | **Form** |
| --- | --- | --- | --- | --- | --- |
| **Actiq (Consumer Medicine Information: Actiq, 2010)** | Fentanyl | Opioid - Synthetic | Analgesic | Used for management of breakthrough pain in chronic conditions | Lozenge or "lollipop" - fast-release |
| **Antenex (Consumer Medicine Information: Antenex, 2008)** | Diazepam | Benzodiazepine | Sedative/Muscle Relaxant |  | Pill - (2mg & 5mg) |
| **Arcoxia (Consumer Medicine Information: Arcoxia, 2011)** | Etoricoxib | NSAID (selective) | Anti-Inflammatory | Prescription medication used for arthritis or gout | Pill - 30mg, 60mg & 120mg |
| **Biodone (Consumer Medicine information: Biodone, 1999)** | Methadone | Opioid - Synthetic | Analgesic | Low cost, long-acting | Same strength as methadone syrup but has fewer additives. It puts less strain on the liver to process and is better for those who have Hepatitis B or Hepatitis C (Frequently Asked Questions: Opioid Pharmacotherapy Treatments). |
| **Cafergot (Consumer Medicine Information: Cafergot, 2008)** | Ergotamine | Ergot Alkaloid | Vasoconstrictor | prescribed for migrane | Pill (1mg ergotamine & 100mg caffeine) & suppositories (2mg ergotamien & 100mg caffeine) |
| **Celebrex (Consumer Medicine Information: Celebrex, 2010)** | Celecoxib | NSAID (selective) | Anti-Inflammatory | Prescription medication used for arthritis or joint injuries | Pill - 100mg & 200mg |
| **Celestone Chronodose (Consumer Medicine Information: Celestone Chronodose, 2011)** | Cortisone | Corticosteroid | Steroid - Anti-Inflammatory | Immunosuppressant that reduces inflammation | Injection (Epidural) |
| **Cicloral (Consumer Medicine Information: Cicloral, 2010)** | Cyclosporin | Disease Modifying Anti-Rheumatic Drug (DMARD) | Immunosuppressant |  | Pill - (25mg, 50mg or 100mg) |
| **Codalgin Plus (Consumer Medicine Information: Codalgin Plus, 2005)** | Codeine | Opioid | Analgesic | Over the counter pain-relief medication that contains an antihistamine with sedative effects. | Pill - contains 500mg Paracetemol, 9.6 mg Codeine & 9.6 mg Doxylamine (antihistamine) |
| **Cortisone (Fadale & Wiggins, 1994)** | Cortisone | Corticosteroid | Steroid - Anti-Inflammatory | Immunosuppressant that reduces inflammation | Pill or Injection |
| **Cymbalta (Consumer Medicine Information: Cymbalta, 2012)** | Duloxetine Hydrochloride | Serotonin & Noradrenaline Reuptake Inhibitors (SNRI) | Anti-Depressant | Also prescribed for nerve pain | Pill - 30mg, 60mg |
| **Di-Gesic (Consumer Medicine Information: Di-Gesic, 2012)** | Dextrapro-poxyphene | Opioid | Analgesic | 2012 - Therapeutic Goods Administration has ordered the de-registration of this drug |  |
| **Dilaudid (Consumer Medicine Informtation: Dilaudid, 2010)** | Hydromorphone | Opioid -Semi-Synthetic | Analgesic | Better speed of onset and lower dependence liability than morphine, but dependence and tolerance can build quickly | instant-release pill taken every 1-4 hours |
| **Dolased (Consumer Medicine Informaiton: Dolased Analgesic, 2004)** | Codeine, Doxylamine, & Paracetemol |  | Analgesic | Over the counter pain-relief medication | Pill (500mg paracetemol, 10mg Codeine, 5.1mg Doxylamine Succinate) |
| **Doloxene (Consumer Medicine Information: Doloxene, 2012)** | Dextrapro-poxyphene | Opioid | Analgesic | 2012 - Therapeutic Goods Administration has ordered the de-registration of this drug |  |
| **Duragesic (Consumer Information: Duragesic, 2011)** | Fentanyl | Opioid - Synthetic | Analgesic | Used for management of chronic pain | Patches - slow-release (48-72 hrs) |
| **Duratram (Consumer Medicine Information: Duratram, 2008)** | Tramadol | Opioid - Synthetic | Analgesic | Acts differently from other opioids - effective against nerve pain |  |
| **Endone (Consumer Medicine Information: Endone, 2010)** | Oxycodone | Opioid - Semi-Synthetic | Analgesic | Can be used to manage chronic pain; can be better tolerated than morphine by some | Immediate-release pill taken every 4-6 hrs to manage breakthrough pain (Analgesia (Pain-Relievers)). |
| **Humira (Consumer Medicine Information: Humira, 20089)** | Adalimumab | Antibody | Antibody | Intended for treatment of arthritis and other inflammatory diseases such as Crohn's disease | Injection |
| **Imigran (Consumer Medicine Information: Imigran, 2007)** | Sumatriptan | Triptan | Serotonin Receptor Agonist | prescribed for migraine headaches | Pill - 50mg & 100mg |
| **Jurnista (Consumer Medicine Information: Jurnista prolonged release tablet , 2010)** | Hydromorphone | Opioid -Semi-Synthetic | Analgesic | Better speed of onset and lower dependence liability than morphine, but dependence and tolerance can build quickly | 24 hour extended release pill |
| **Kapanol (Consumer Medicine Information: Kapanol, 2006)** | Morphine | Opioid | Analgesic | Often prescribed for chronic severe pain | Available in both 12 hr or 24 hour extended-release capsules |
| **Ledertrexate (Patient Information on Methotrexate, 2008)** | Methotrexate | Disease Modifying Anti-Rheumatic Drug (DMARD) | Immunosuppressant | Intended for treatment of rheumatoid arthritis and lupus | Pill - once a week (2.5mg or 10mg) |
| **Lyrica (Consumer Medicine Information: Lyrica, 2011)** | Pregabalin | Anti-Convulsant | Anti-Convulsant | Used to treat neuropathic pain | Pill - 25mg, 75mg, 150mg, 300mg |
| **Maxalt (Consumer Medicine Information: Maxalt Wafers, 2012)** | Rizatriptan | Triptan | Serotonin Receptor Agonist | prescribed for migraine headaches | Wafer that is dissolved on tongue - 5mg or 10mg |
| **Mersyndol (Consumer Medicine Information: Mersyndol, 2007)** | Codeine | Opioid | Analgesic | Over the counter pain-relief medication | Pill - contains 500mg Paracetmol & 9.6 mg Codeine |
| **Methadone syrup (Consumer Medicine Information: Methadone Syrup, 2007)** | Methadone | Opioid - Synthetic | Analgesic | Low cost, long-acting liquid, considered more effective against neuropathic pain |  |
| **Methoblastin (Patient Information on Methotrexate, 2008)** | Methotrexate | Disease Modifying Anti-Rheumatic Drug (DMARD) | Immunosuppressant | Intended for treatment of rheumatoid arthritis and lupus | Pill - once a week (2.5mg or 10mg) |
| **Mobic (Consumer Medicine Information: Mobic, 2009)** | Meloxicam | NSAID | Anti-Inflammatory | Prescription medication used to treat arthritis | Pill - 7.5mg & 15mg |
| **MSContin (Consumer Medicine Information: MS Contin, 2010)** | Morphine | Opioid | Analgesic | Often prescribed for chronic severe pain | Pills – extended-release taken every 12 hours. Available in seven strengths: 5, 10, 15, 30, 60, 100, 200 mg. |
| **Naprosyn (Consumer Medicine Information: Naprosyn, 2008)** | Naproxen | NSAID | Anti-Inflammatory | Prescription only pain relief often recommended for migraine or arthritis | Pill - 250mg & 500mg, also in 24hr slow release tablets |
| **Naramig (Consumer Medicine Information: Naramig, 2007)** | Naratriptan | Triptan | Serotonin Receptor Agonist | Can be prescribed for relief of migraine headaches | Pill - 2.5mg |
| **Neoral (Consumer Medicine Information: Neoral, 2010)** | Cyclosporin | Disease Modifying Anti-Rheumatic Drug (DMARD) | Immunosuppressant | Prescription medication used to treat rheumatoid arthritis | Either solution or pill (25mg, 50mg, & 100mg) |
| **Normison (Consumer Medicine Information: Normison, 2009)** | Temazepam | Benzodiazepine | Sedative/Hypnotic/Muscle Relaxant | Can be prescribed for chronic back pain | Pill - 10 mg |
| **Norspan (Consumer Medicine Information: Norspan, 2009)** | Bupenorphine | Opioid - Semi-Synthetic | Analgesic | Controlled release of medication to client | Patch - slow release (7 days) |
| **Numorphan (Oxymorphone)** | Oxymorphone | Opioid - Semi-Synthetic | Analgesic | Not available in Australia - can be imported |  |
| **Numorphone (Oxymorphone)** | Oxymorphone | Opioid - Semi-Synthetic | Analgesic | Not available in Australia - can be imported |  |
| **Nurofen (Pain relievers explained, 2012)** | Ibuprofen | NSAID | Anti-Inflammatory | Over the counter pain-relief medication | Pill, Gel, Heat Patches |
| **Nurofen Plus (Consumer Medicine Information: Nurofen Plus, 2009)** | Codeine | Opioid | Analgesic | Over the counter pain-relief medication | Pill - contains 200mg Ibuprofen & 12.8 mg Codeine |
| **Opana (Opana Consumer Information, 2012)** | Oxymorphone | Opioid - Semi-Synthetic | Analgesic | Not available in Australia - can be imported |  |
| **Ordine (Consumer Medicine Information: Ordine, 2010)** | Morphine | Opioid | Analgesic | Often prescribed for chronic severe pain when other treatments are not effective. | Fast-acting Oral liquid solution: 1,2,5,10 mg (every four hours) |
| **Orencia (Consumer Medicine Information: Orencia, 2012)** | Abatacept | Disease Modifying Anti-Rheumatic Drug (DMARD) | Immunosuppressant | Used for rheumatoid arthritis when there is an inadequate response to other drugs. | Either slow infusion into vein (30 minutes) or injection |
| **Orudis (Consumer Medicine Information: Orudis, 2011)** | Ketoprofen | NSAID | Anti-Inflammatory | Prescription only pain relief often recommended for arthritis or other musculo-skeletal inflammation | Topical Gel (750mg/30g or 1.5g/60g) or capsules (200 mg) |
| **Oruvail (Consumer Medicine Information: Oruvail, 2011)** | Ketoprofen | NSAID | Anti-Inflammatory | Prescription only pain reliever often used to reduce pain & inflamation with arthritis | Pill - 200mg |
| **OxyContin (Consumer Medicine Information: OxyContin Tablets, 2010)** | Oxycodone | Opioid - Semi-Synthetic | Analgesic | Can be used to manage chronic pain; can be better tolerated than morphine | Extended-Release Pill (every 12 hours) – 5, 10, 15, 20, 30, 40, 80 mg |
| **OxyNorm (Consumer Medicine Information: OxyNorm, 2010)** | Oxycodone | Opioid - Semi-Synthetic | Analgesic | Can be used to manage chronic pain; can be better tolerated than morphine | Short-acting pill (every 4-6 hrs) - 5, 10, 20 mg |
| **Panadeine (Consumer Medicine Information: Panadeine, 2009)** | Codeine | Opioid | Analgesic | Over the counter pain-relief medication | Pill, rapid soluble, contains 500mg Paracetemol & 8mg Codeine |
| **Panadeine Extra (Consumer Medicine Information: Panadeine Extra, 2009)** | Codeine | Opioid | Analgesic | Strongest over-the counter medication containing codeine; contains paracetamol & codeine | Pill - contains 500mg Paracetemol & 15 mg Codeine |
| **Panadeine Forte (Consumer Medicine Information: Panadeine Forte, 2007)** | Codeine | Opioid | Analgesic | Only available with prescription | Pill - contains 500mg Paracetemol & 30 mg Codeine |
| **Panadol Extra (Panadol Extra Product Information, 2009)** | Paracetemol | NSAID | Anti-Inflammatory | Over the counter pain-relief medication recommended for headaches, arthritis, and other pain. Caffeine helps with the absorption of the paracetamol. | Pill - contains 500mg Paracetemol & 65mg Caffeine |
| **Panafcort (Consumer Medicine Information: Panafcort, 2010)** | Prednisone | Corticosteroid | Steroid - Anti-Inflammatory | Reduce swelling and pain in joints/other organs | Pill - available in 1mg, 5mg, and 25mg doses |
| **Panafcortelone (Consumer Medicine Information: Panafcortelone, 2009)** | Prednisolone | Corticosteroid | Steroid - Anti-Inflammatory | Reduce swelling and pain in joints/other organs | Pill - available in 1mg, 5mg, and 25mg doses |
| **Paradex (Consumer Medicine Information: Paradex, 2010)** | Dextropro-poxyphene | Opioid | Analgesic | 2012 - Therapeutic Goods Administration has ordered the de-registration of this drug in Australia (Update on TGA decision to cancel prescription pain-killers, 2012) | Pill – 32.5mg dextropropoxyphene & 325mg Paracetamol |
| **Pethidine (Consumer Medicine Information: Pethidine, 2008)** | Pethidine | Opioid - Synthetic | Analgesic | High Toxicity and Fast release has reduced popularity for prescription for chronic pain. Higher reported euphoric sensations than other opiates. | Available by white tablet or medically-supervised injection |
| **Physeptone (Consumer Medicine Information: Physeptone Tablets, 2011)** | Methadone | Opioid - Synthetic | Analgesic | Methadone in tablet form - more likely to be subscribed for relief of chronic pain |  |
| **Plaquenil (Consumer Medicine Information: Plaquenil, 2006)** | Hydroxychloroquine Sulfate | Anti-Malarial | Anti-Inflammatory | Prescription only - often prescribed for arthritis or lupus | Pill - 200mg |
| **Predsolone (Consumer Medicine Information: Predsolone, 2009)** | Prednisolone | Corticosteroid | Steroid - Anti-Inflammatory | Used to reduce symptoms of inflammation, including pain in arthritis. | Pill - available in 1mg dose |
| **Predsone (Consumer Medicine Information: Predsone, 2009)** | Prednisone | Corticosteroid | Steroid - Anti-Inflammatory | Used to reduce symptoms of inflammation, including pain in arthritis. | Pill - available in 1mg dose |
| **Rapifen (Consumer Medicine Information: Rapifen, 2011)** | Alfentanil | Opioid - Synthetic | Analgesic | Potent, short-acting, often used for anaesthesia | Injection |
| **Relpax (Consumer Medicine Information: Relpax, 2012)** | Eletriptan | Triptan | Serotonin Receptor Agonist | prescribed for relief of migraine headaches | Pill - 40mg & 80 mg |
| **Sandimmun (Consumer Medicine Information: Sandimmun, 2011)** | Cyclosporin | Disease Modifying Anti-Rheumatic Drug (DMARD) | Immunosuppressant | Can be prescribed to reduce inflammation in rheumatoid arthritis | Either solution or pill (25mg, 50mg, & 100mg) |
| **Solone (Consumer Medicine Information: Solone, 1997)** | Prednisolone | Corticosteroid | Steroid - Anti-Inflammatory |  | Pill - available in 5mg & 25mg doses |
| **Sone (Consumer Medicine Information: Sone, 2001)** | Prednisone | Corticosteroid | Steroid - Anti-Inflammatory |  | Pill - available in 5mg & 25mg doses |
| **Suboxone (Consumer Medicine Information: Suboxone, 2010)** | Bupenorphine | Opioid - Semi-Synthetic | Analgesic | Used as both analgesic and opioid substitution treatment | Pill or Film form - contains opioid antagonist Naloxone to discourage intravenous drug use |
| **Subutex (PBS - Subutex (buprenorphine), 2003)** | Bupenorphine | Opioid - Semi-Synthetic | Analgesic | Used as both analgesic and opioid substitution treatment | Pill - administered sublingually |
| **Sumagran (Consumer Medicine Information: Sumagran, 2007)** | Sumatriptan | Triptan | Serotonin Receptor Agonist | prescribed for relief of migraine headaches | Pill - 50mg & 100mg |
| **Sumatab (Consumer Medicine Information: Sumatab, 2009)** | Sumatriptan | Triptan | Serotonin Receptor Agonist | prescribed for migraine headaches | Pill - 50mg & 100mg |
| **Targin (Consumer Medicine Information: Targin, 2011)** | Oxycodone | Opioid - Semi-Synthetic | Analgesic | Can be used to manage chronic pain; contains naloxone which can block some of the effects of opioids in the gut, such as constipation. | Pill – 2.5, 5, 10, 20 mg |
| **Temgesic (Consumer Medicine Information: Temgesic, 2008)** | Bupenorphine | Opioid - Semi-Synthetic | Analgesic | Used as both analgesic and opioid substitution treatment | Pill - administered sublingually, or injection |
| **Tramal (Consumer Medicine Information: Tramal, 2006)** | Tramadol | Opioid - Synthetic | Analgesic | Acts differently from other opioids - effective against nerve pain |  |
| **Valium (Consumer Medicine Information: Valium, 2010)** | Diazepam | Benzodiazepine | Sedative/Muscle Relaxant | Can be used as a muscle relaxant for back pain | Pill - (2mg & 5mg) |
| **Vicodin** | Hydrocodone | Opioid - Semi-Synthetic | Analgesic | Not available in Australia - can be imported | Hydrocodone is similar to codeine. Combined with paracetamol. |
| **Xanax (Consumer Medicine Information: Xanax, 2011)** | Alprazolam | Benzodiazepine | Sedative/Muscle Relaxant | Can be used as a muscle relaxant for back pain |  |
| **Xylocaine (Consumer Medicine Information: Xylocaine, March)** | Lignocaine Hydrochloride | Local Anaesthetic | Anaesthetic | can be used to soothe peripheral neuropathic pain | Injection or Jelly 20mg/g |
| **Zomig (Consumer Medicine information: Zomig, 2006)** | Zolmitriptan | Triptan | Serotonin Receptor Agonist | prescribed for relief of migraine headaches | Pill (2.5mg or 5mg) or orally dispersible tablets |
| **Zydol (Consumer Medicine Information: Zydol, 2011)** | Tramadol | Opioid - Synthetic | Analgesic | Acts differently from other opioids - effective against nerve pain | Sustained release pills (1-2 per day) – 50, 100, 150 & 200 mg |
